# Preparation and Characterization of Microencapsulated Phase Change Materials for Use in Building Applications

**DOI:** 10.3390/ma9010011

**Published:** 2015-12-26

**Authors:** Jessica Giro-Paloma, Refat Al-Shannaq, Ana Inés Fernández, Mohammed M. Farid

**Affiliations:** 1 Department of Materials Science and Metallurgical Engineering, Faculty of Chemistry, Universitat de Barcelona, C/Martí i Franquès, Barcelona 1. 08028, Spain; jessicagiro@ub.edu (J.G.-P.); ana_inesfernandez@ub.edu (A.I.F.); 2 Department of Chemical and Materials Engineering, University of Auckland, Private Bag 92019, 20 Symonds Street, Auckland 1142, New Zealand; rals187@aucklanduni.ac.nz

**Keywords:** microencapsulated phase change material, volatile organic compounds, nano-indentation, differential scanning calorimetry

## Abstract

A method for preparing and characterizing microencapsulated phase change materials (MPCM) was developed. A comparison with a commercial MPCM is also presented. Both MPCM contained paraffin wax as PCM with acrylic shell. The melting temperature of the PCM was around 21 °C, suitable for building applications. The M-2 (our laboratory made sample) and Micronal^®^ DS 5008 X (BASF) samples were characterized using SEM, DSC, nano-indentation technique, and Gas Chromatography/Mass spectrometry (GC-MS). Both samples presented a 6 μm average size and a spherical shape. Thermal energy storage (TES) capacities were 111.73 J·g^−1^ and 99.3 J·g^−1^ for M-2 and Micronal^®^ DS 5008 X, respectively. Mechanical characterization of the samples was performed by nano-indentation technique in order to determine the elastic modulus (*E*), load at maximum displacement (*P_m_*), and displacement at maximum load (*h_m_*), concluding that M-2 presented slightly better mechanical properties. Finally, an important parameter for considering use in buildings is the release of volatile organic compounds (VOC’s). This characteristic was studied at 65 °C by CG-MS. Both samples showed VOC’s emission after 10 min of heating, however peaks intensity of VOC’s generated from M-2 microcapsules showed a lower concentration than Micronal^®^ DS 5008 X.

## 1. Introduction

Thermal energy storage (TES) using phase change materials (PCM) has shown a significant increased attention because of its important role on energy conservation in buildings [1,2,3,4]. PCM can be used for TES in buildings [5] either in passive [6] or active systems [7,8,9], aiming to improve the thermal managements of these buildings. In most of the applications, PCM were used either in macroencapsulated [10,11] or microencapsulated [12,13,14] forms, for heating [15], air-conditioning [16], ventilation [17], refrigeration [18], and heat exchangers [19] for building applications [3,20,21,22].

Microencapsulation process is defined as a technique in which small particles or droplet are enclosed by a coating, or surrounded in a homogeneous or heterogeneous matrix, to provide microcapsules (1–100 μm). For this reason, the microencapsulated phase change materials (MPCM) are composed of PCM as a core and a polymer as a shell used to preserve the spherical shape of the microcapsule and avoid PCM leakage during phase change [12,23]. The use of MPCM in buildings [24,25,26,27] can decrease daily inner temperature fluctuation during summer and winter [28]. The suitability of the shell used in encapsulating specific core PCM is a key issue in order to ensure proper thermal performance of the MPCM [13,29,30], especially in preventing PCM leakage when it melts. Additionally, MPCM can be easily incorporated in gypsum board [31,32], plaster [33], and concrete [34] used in buildings.

The complete characterization of materials used in indoor environments like MPCM is very important. The exposure to chemical compounds could cause health problems (nausea; dry skin; eye, nose or throat irritations; headache; irritated eyes; dizziness; difficulty in concentrating; psychological stress) in indoor environments [35,36,37,38] (buildings, for example [39,40,41,42,43,44,45,46,47]) or outdoor environments [48]. These problems are known as sick building syndrome (SBS) [37,49,50,51]. Volatile organic compounds (VOC’s) are defined as any organic compound having an initial boiling point less than or equal to 250 °C at a standard pressure of 101.3 kPa [52]. VOC’s are one of the most important groups of trace contaminants in the atmosphere known for its photochemical, toxic, and radioactive effects. For this reason there are some studies, guides [53,54], and database [55,56] related to this effect. Formaldehyde [39,46,57] and benzene [58] are some of the most studied pollutants since they are classified in Group 1 of human carcinogens by the IARC 2004 (International Agency for Research on Cancer). Other chemicals known for their health hazard are acetaldehyde, toluene, and xylenes [59]. By this way, VOC’s evaluation of the outdoor and indoor air quality has been evaluated [28,29,30,31] in materials for buildings like gypsum base PCM composites [60] but it has not been reported for building materials containing MPCM. For this reason, the characterization of VOC’s of MPCM is an important contribution to the state of the art of the environmental properties of MPCM.

The main purpose of this research is to develop, prepare, characterize, study, and compare thermal and mechanical properties of microcapsules containing organic PCM in order to assess their suitability for use in buildings and other applications. The samples under study are commercial MPCM (Micronal^®^ DS 5008 manufactured by BASF, Berlin, Germany) and a laboratory prepared one by us (M-2). Micronal^®^ DS 5008 sample has been used extensively in concrete, gypsum, lime plaster, and gypsum plaster, without being fully characterized for fire hazards. The comparison includes fire retardancy and gas emission released to environment from upon fire. It is important to establish a characterization methodology, which will include both volatile emission measurements and nano-indentation technique to measure the shell mechanical strength of the microcapsules. This is very important issue in the selection of PCM products, especially for use in building application. PCM microcapsules should have high phase change enthalpy, uniform spherical shape, acceptable thermal stability, good mechanical properties, and low release of hazardous gases in the form of volatile organic compounds.

## 2. Materials and Methods

### 2.1. Materials

The chemical preparation of microcapsules required the following reagents:

- Shell: Methyl methacrylate (MMA) (99%, contains ≤ 30 ppm monomethyl ether hydroquinone (MEHQ) as inhibitor, Sigma Aldrich, Auckland, New Zealand) and pentaerythritol tetraacrylate (PETRA) (contains 350 ppm (MEHQ), Sigma Aldrich, Auckland, New Zealand) were used as a monomer and cross-linking agent respectively in order to obtain proper shells for MPCM.
- Free radical thermal initiator: Luperox^®^ A75, Benzoyl peroxide (BPO) (75%, contains 25% water, Sigma Aldrich, Auckland, New Zealand) was used as free radical thermal initiator.
- Surfactants: Polyvinyl alcohol (PVA) (M_w_ 85,000–124,000, Sigma Aldrich, Auckland, New Zealand) and sodium dodecyl sulfate (SDS) (BioXtra, 99%, Sigma Aldrich, Auckland, New Zealand) were used as a non-ionic and ionic surfactant, respectively.
- PCM: a commercial paraffinic PCM, Rubitherm^®^ RT 21 (T_m_ = 21 °C, ΔH_m_ = 135 J·g^−1^, Rubitherm^®^ Technologies GmbH, Berlin, Germany) was used.

The bulk density of M-2 microcapsules is 0.496 g·mL^−1^. The commercial MPCM, Micronal^®^ DS 5008 X (BASF^®^), was also selected for characterization and was compared with the microcapsules produced in this work. This sample is also composed by an acrylate shell and organic PCM in the core [13], and its bulk density is 0.445 g·mL^−1^.

### 2.2. Synthesis of PCMs Microcapsules

#### 2.2.1. Emulsification

A standard procedure was used as reported elsewhere [61]. Wherein, an aqueous solution of surface-active agent (called aqueous phase) and a mixture of MMA, PETRA, BPO, and PCM (called organic phase) were prepared separately. The organic phase was added to the aqueous phase and emulsified mechanically using a high shear mixer (Silverson L5M-A laboratory Mixer, Silverson LTD, East Longmeadow, MA, USA). A stirring rate of 3000 rpm for 5 min was chosen to achieve the required emulsification.

#### 2.2.2. Polymerization

The produced emulsion was transferred to a 2-L four-neck glass reactor (LR-2.ST laboratory reactor-IKA-Werke, Gmbh@Co.KG, Staufen, Germany) consisting of EUROSTAR 200 control P4, Anchor stirrer LR 2000.1, HBR 4 digital heating bath. The agitation speed was set at approximately 300 rpm, and the temperature of the water bath was maintained at 70 °C for 2 h, and then adjusted to 85 °C for another 4 h. The water bath was then switched off and allowed to cool down naturally to room temperature. After cooling, the suspension of PCM microcapsules was transferred to a clean glass beaker and washed three times with distilled water to remove the unreacted monomers and the non-encapsulated PCM. The separated microcapsules were spread on a tray and placed in an oven at 50 °C for 48 h for drying. The dried microcapsules were then collected for testing.

## 3. Characterization of Microcapsules

### 3.1. Scanning Electron Microscopy (SEM)

To study the shape and size of microcapsules SEM was used (a FEI Quanta 200 FEG-Field Emission Gun with an EDS Detector SiLi (Lithium drifted) with a Super Ultra-Thin window, FEI Company, Hillsboro, OR, USA). The sputter coater used was a Quorum Q150RS (FEI Company, Hillsboro, OR, USA), and it is designed to give an appropriate thin, slight metal coating proper for SEM observation, using Pt as a target. The coating thickness and uniformity of the sample depends on different factors: distance between sample and target, topography of the sample, and affinity of the material with the metal coating.

### 3.2. Differential Scanning Calorimetry (DSC)

Phase change properties of the fabricated PCM microcapsules and the pure PCM (such as melting and solidification temperatures and their phase change enthalpies) were determined using a SHIMADZU DSC-60 differential scanning calorimeter (Shimadzu Company, Kyoto, Japan). The measurements were performed by varying the temperature between −15 °C and 40 °C with heating and cooling rate of 3 °C·min^−1^. Each sample was analyzed for three times and the average was taken. Consequently, the percentage PCM encapsulated can be determined using DSC results and the following Equation (1) [62,63]. The mass content obtained by DSC measurements does not provide accurate measure of the core mass content. Equation (1), which was used to estimate the mass content from DSC measurements, does not take in account the sensible heat of coating materials. The TGA method provides more accurate measure of the core material mass content than DSC. In our previous publication [63] the core material mass content of sample M-2 obtained by TGA is 77.5 wt%, which is less than the one obtained by DSC.

(1) % PCM in microcapsules by mass=ΔHmicrocapsulesΔHPure PCM×100%

where *ΔH_microcapsules_* (J·g^−1^) is the latent heat of the microcapsule containing PCM; and *ΔH_purePCM_* (J·g^−1^) is the latent heat of pure PCM (before encapsulation). In Equation (1), it is assumed that the latent heat of the microcapsule without PCM is zero, which is true if phase change does not occur in the shell does.

### 3.3. Nano-Indentation Technique

**Figure 1:**
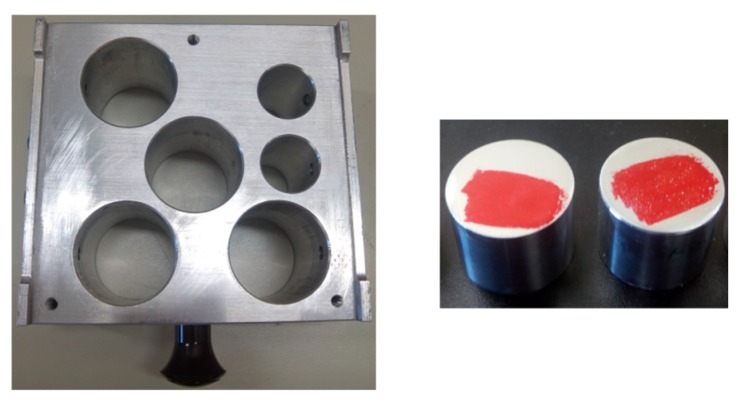
Holder and aluminum stubs with the sample over the red glue.

To characterize the mechanical properties of M-2 and commercial Micronal^®^ DS 5008 X samples, a nano-indentation technique was used. Nano-indentation is identified as an appropriate technique to test the strength of individual microcapsules [64]. MTS Nano Indenter XP (MTS Company, Eden Prairie, MN, USA) was the instrument used. Aluminium stubs of 20 mm height and 30 mm diameter were needed to stick the samples at the top to characterize them using a red glue to stick the samples as shown in Figure 1. The instrument parameters were set the same for the two studied samples for a more accurate comparison.

There are some required inputs to set before starting the experiments: strain rate target of 0.05 s^−1^, allowable drift rate of 0.05 nm·s^−1^, a Poissons’ ratio of 0.18 for the tip indenter [65,66] a peak hold time of 10 s, a surface speed of 10 nm·s^-1^, 25% of surface approach sensitivity, 90% to unload, an approach distance to store of 1000 nm, a surface approach distance of 1000 nm, and finally, a depth limit of 5000 nm.

To determine the elastic modulus (*E*) of the studied samples, a Berkovich tip TB-13288 (Micro Star Technologies, Huntsville, TX, USA) was used. The use of nano-indentation for the characterization of mechanical properties of materials has been extensively studied by several authors. Oliver and Pharr developed extensively the methodology for characterizing ceramic materials [66,67]. They described the typical load *vs*. displacement curve, where increasing the load (*P*) increases the displacement (*h*) until reaching a maximum load (*P_max_*) and a maximum displacement (*h_max_*). Following that, the indenter is removed out of the material (unloading section), the load will be zero, and the final displacement (*h_f_*) will be measured. After that, the *E* value for each sample can be calculated. Hochstetter *et al*. [68] presented results for glassy polymers and Giro-Paloma *et al*. [69] compared both methodologies using continuous stiffness measurement (CSM) by applying a small oscillation to the quasi-static component of loading using different thermoplastics suitable as containers for PCM. They concluded that Loubet’s method produce lower values of *H* and *E* because it uses a dynamic approach for stiffness measurements and the contact depth is larger due to the contribution of the apparent tip effect. In the light of these findings, it was concluded that Loubet’s method should be used only with polymeric materials having a low viscous character (*T_g_* > *T_measurement_*). Additionally, in depth-sensing indentation (DSI), which is the mode used in this paper, load is applied as a function of penetration depth during the loading and unloading cycle, as described by Fischer-Cripps [70]:

- Hardness (*H*) is defined as the maximum indentation load divided by the cross-sectional area of the indenter specified at the maximum indentation depth (*A*(*h_m_*)).
- Load (*P_m_*) at maximum displacement (mN): It is the load recorded at the maximum load, which occurs when sample fails.
- Elastic modulus (*E*) is evaluated following Equations (2) and (3) from the nano-indentation test using the maximum indentation load (*P_m_*) and the depth sensing indentation. Hardness (*H*), elastic work (*W_e_*), and total work (*W*) can be calculated by integrating the areas under the indentation unloading. *W_e_* and *W* are the elastic work and total work, which are equal to the areas under the unloading and loading curves, respectively which is correlated with *E* and *H* through the function ψ described in [71]. WeW value is independent of the degree of work-hardening behavior [66].
  (2) H=PmA(hm)
  (3) E=Hψ(WeW)
- Displacement (*h_m_*) at maximum load (nm) is a measure of the extent the tip penetrates into the material.

### 3.4. Emission of Volatile Organic Compounds (VOC’s)

A GC-17A Gas chromatograph Shimadzu (Shimadzu Corporation, Kyoto, Japan) coupled to GCMS-QP5000 Gas chromatograph/Mass Spectrometer Shimadzu (Shimadzu Corporation, Kyoto, Japan) was used to characterize the VOC’s emissions from each sample. A calibration was performed for each pure compound: C_14_H_30_, C_15_H_32_, C_16_H_34_, C_17_H_36_, C_18_H_38_, C_20_H_42_, C_22_H_46_, and C_24_H_50_, at different temperatures: 25 °C, 35 °C, 45 °C, 55 °C, and 65 °C. A total of 40 experiments for calibration were executed. When the calibration was completed, the same procedure was performed for the two studied samples: M-2 and Micronal^®^ DS 5008 X.

Each sample was independently located inside a crystal vial HS of 50 mL capacity. The vials were submerged during 30 min in a water bath until reaching the required temperature. Later on, a fibber solid-phase microextraction (SPME) holder with lot number P268618D 57330-U, provided by Supelco (Sigma-Aldrich Corporation, St. Louis, MO, USA), was punctured on the top of the silicone cap. Following that, 10 min desorption was applied. The temperature inside the device was 60 °C during 2 min. After that, a ramp of 15 °C·min^−1^ was programmed.

A HP-5MS (1553434H) (Agilent Technologies, Santa Clara, CA, USA) was the column used. Its thickness, length and diameter were 0.5 μm, 30 m, and 0.32 cm, respectively. Additionally, the injection and interface temperature were 200 °C and 280 °C, respectively inside the gas chromatographer. There were more parameters to take into account, such as inlet pressure: 1 kPa, flow: 1.1 mL·min^−1^, lineal velocity: 38.7 cm·s^−1^, split ratio between peaks of 20, and finally total flow for the He gas of 23.1 mL·min^−1^. On the other hand, the mass spectrometer m/z values are from 35 to 350. Moreover, the solvent cut time was 0.5 min.

## 4. Results and Discussion

### 4.1. Characterization of MPCM Shape, Size, and Morphology

**Figure 2:**
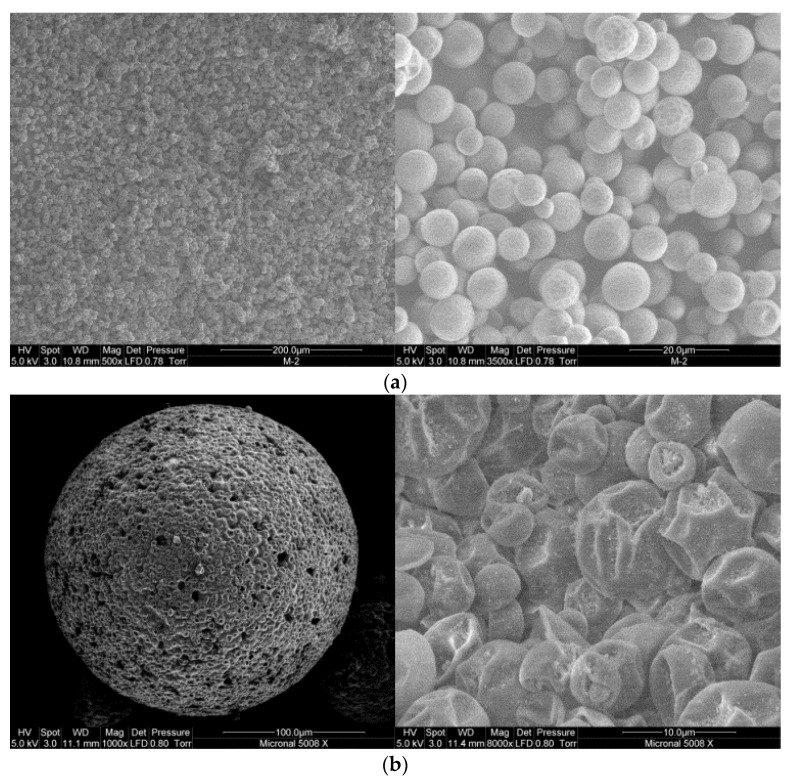
SEM images for the studied samples: (**a**) M-2 (magnification: ×500 left and ×3500 right); (**b**) Micronal^®^ DS 5008 X (magnification: ×1000 left and ×8000 right).

SEM images for the two studied samples are shown in Figure 2. M-2 microcapsules morphology appears to be compact and with smooth surface as shown in Figure 2a. Furthermore, their size is around 6 μm and has a spherical shape. On the other hand, commercial Micronal^®^ DS 5008 X looks made of a big sphere (around 150 μm) composed of hundreds of other small microcapsules (the ones which contain the PCM) with 6 μm in size, approximately as shown in Figure 2b. As Figure 2 shows, the 6 μm microcapsules of Micronal^®^ DS 5008 X samples are deformed, which is probably due to the process of agglomerating of these microcapsules to form larger particle of 150 μm, which probably has been made for ease of handling. Accelerated thermal cycling experiments of PCM microcapsules (M-2 containing RT-58 sample) were performed in a controlled heating/cooling water bath at temperatures cycling between 2 and 40 °C in our previous publication [63]. The results showed that slight changes in phase transition temperatures of the PCM microcapsules (M-2 containing RT58 sample) after 2000 cycles. The latent heat of M-2 sample (based on DSC measurements) showed only a minor change of 2% after 2000 cycles. Furthermore, SEM images showed that the capsule shape remained spherical and no shell cracks were observed at the end of 2000 cycles.

### 4.2. Thermophysical Properties of MPCM

**Figure 3:**
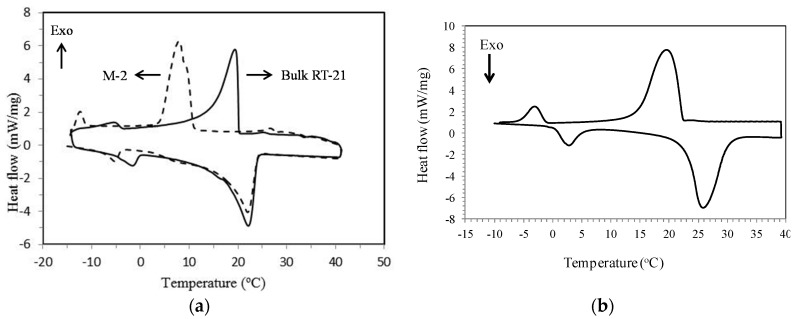
DSC results for: (**a**) M-2; and (**b**) Micronal^®^ DS 5008 X.

**Figure 4:**
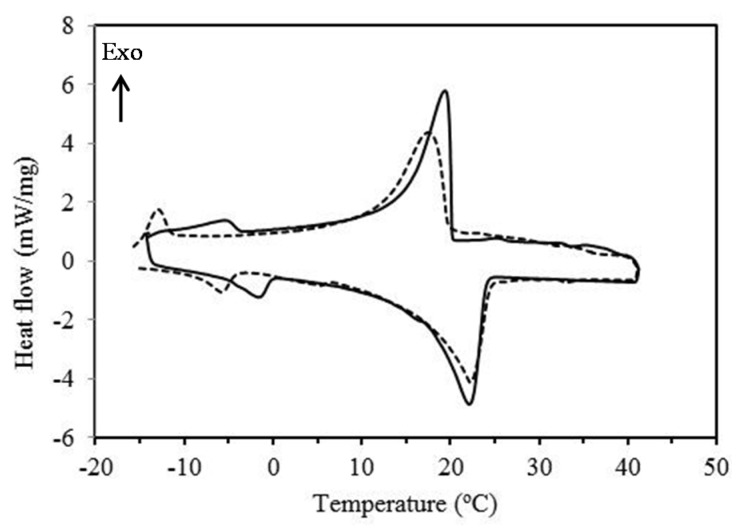
DSC curves of the bulk RT-21 (solid line) and M-2 microcapsules containing nucleating agent, RT-58 (dotted line).

Thermal properties of M-2 and Micronal^®^ DS 5008 X in terms of phase change enthalpies and phase change temperatures were investigated using DSC as shown in Figure 3. Based on DSC measurements, the % mass of the PCM for M-2 sample is 81.7 wt% and for Micronal^®^ DS 5008 X is 78.1 wt%. The DSC results show that the thermal energy storage capacity of the M-2 is 113.8 J·g^−1^ which corresponds to 85 wt% of RT-21 encapsulation. The melting temperature of the RT-21 in M-2 microcapsules is similar to that of the bulk RT-21. In contrast, the solidification temperature of the PCM microcapsules was about 14 °C lower than that of the bulk RT-21 (super-cooling phenomena) as previously reported [72] (see Table 1). The supercooling of PCM in microcapsules has also been reported by Qiu *et al*. [73]. The increase of the degree of super-cooling of RT-21 microcapsules could be attributed either to the decrease in the amount of RT-21 nuclei inside each microcapsule compared to the bulk RT-21 [74] or due to formation of vacuum pockets space inside the microcapsules [75]. To reduce the supercooling of PCM microcapsules, additives were mixed with the PCM prior encapsulation to act as a nucleating agent. These nucleating agents are usually materials with a similar crystal structure as the solid PCM which allow nucleation at their surface but have a higher melting temperature. Figure 4 shows the DSC curve for the microcapsules containing nucleating agent. Commercial RT-58 (paraffin) with peak melting temperatures of 58 °C was used as nucleating agent. The degree of supercooling has been reduced dramatically as reported in Table 1 and shown in Figure 4.

**Table 1:** Thermophysical properties of fabricated M-2 and commercial Micronal^®^ DS 5008 X.

Status	Transition Temperatures and Heat of Fusion	M-2	RT-21	Micronal^®^ DS 5008 X	M-2 Containing RT-58
Heating	T_onset_ (°C)	16.7	16.5	22.1	16.1
	T_peak_ (°C)	22.0	22.1	20.3	22.3
	T_endset_ (°C)	24.0	23.9	26.3	24.3
	ΔH (J·g^−1^)	113.9	132.0	99.9	110.4
Cooling	T_onset_ (°C)	10.9	20.2	22.5	19.8
	T_peak_ (°C)	7.9	19.4	24.2	17.5
	T_endset_ (°C)	4.2	14.5	17.5	11.4
	ΔH (J·g^−1^)	111.9	132.5	103.5	108.3

### 4.3. Mechanical Properties of MPCM

Results of nano-indentation technique by DSI for the two samples were summarized in Table 2. Ten tests were performed for each sample, but some results were discarded because of the dispersion attributed to indentations in the edge of the microcapsules. For this reason, six results were finally selected. From these measurements the mean value and standard deviation of the elastic modulus (*E*) were calculated for each sample. These results show that M-2 microcapsules are stiffer than Micronal^®^ DS 5008 X ones.

**Table 2:** Elastic modulus results of M-2 and Micronal^®^ DS 5008 X.

Mechanical Property	M-2	Micronal^®^ DS 5008 X
*E* (GPa)	1.89	0.15
	1.04	0.19
	1.16	0.17
	1.68	0.24
	1.38	0.22
	1.9	0.28
Mean	1.51	0.21
Standard Deviation	0.37	0.05

**Figure 5:**
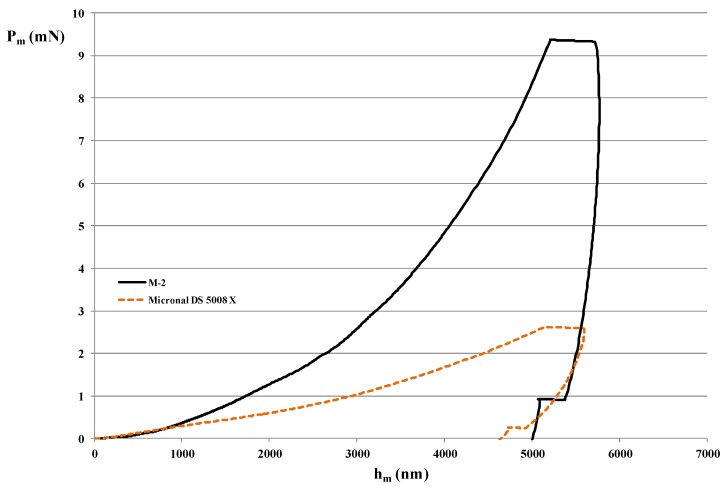
*h_m_ vs. P_m_*, comparison of the samples under study.

Figure 5 is a representation of the *P-h* curves for the M-2 and Micronal^®^ respectively. The measured typical drift behavior can be observed on the plateau at the end of the unloaded curve. It can be concluded that the mechanical response for M-2 is better than Micronal^®^ DS 5008 X as M-2 microcapsules are more rigid and show higher strength. This fact can be attributed to various factors such as the differences in the encapsulation ratio between samples, shell thickness, type of polymers, as well as degree of polymerization of the shells. These results should be compared when mixing these MPCM in a real system, such us mixing them with a cement-based matrix or gypsum.

### 4.4. Characterization of Volatile Organic Compounds (VOCs)

The results of VOCs’ short-term release from the two studied samples are shown in Table 3. Each peak in the GC spectra was given at a certain retention time (*t_r_*, in minutes), which was related to a specific compound, according to the calibration conducted in this work.

**Table 3:** VOC emission results for M-2 and Micronal^®^ DS 5008 X samples.

Temperature	M-2		Micronal^®^ DS 5008 X	
	t_r_ (min)	Compound	t_r_ (min)	Compound
25 °C	No signal		No signal	
35 °C	No signal		No signal	
45 °C	No signal		11.88	C_17_H_36_
			12.66	C_18_H_38_
55 °C	No signal		11.88	C_17_H_36_
			12.65	C_18_H_38_
65 °C	9.25	C_14_H_30_	-	-
	10.18	C_15_H_32_	-	-
	11.06	C_16_H_34_	-	-
	11.91	C_17_H_36_	11.85	C_17_H_36_
	12.68	C_18_H_38_	12.63	C_18_H_38_

**Figure 6:**
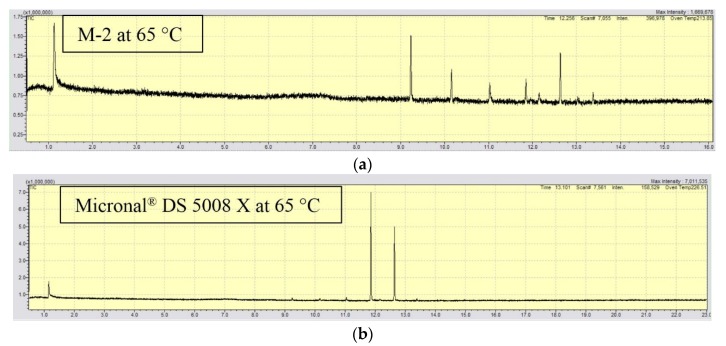
Revealed peaks for the GC/MS analysis for: (**a**) M-2 and; (**b**) Micronal^®^ DS 5008 X samples at 65 °C.

It may be observed from the results that some emissions from Micronal^®^ DS 5008 X sample after 10 min exposure at 45 °C and 55 °C were detected while nothing was detected from M-2 microcapsules. After 10 min exposure at 65 °C, both samples release volatile compounds, but with much lower level for M-2 microcapsules in comparison with Micronal^®^ DS 5008 X. This is confirmed by the high intensity peak for Micronal^®^ DS 5008 X comparing both figures in Figure 6. The multiple peaks for M-2 show the presence of tetradecane, pentacosane, hexadecane, heptadecane, and octadecane in the original PCM, which is RT-21, while only two peaks were observed for the PCM used in Micronal^®^ DS 5008 X, indicating that the latter is of higher purity. Although the results for the short-term emissions are relevant, the long term time-dependent release should be studied in a system after prolonged exhibition to evaluate the VOCs’ emissions in real conditions.

## 5. Conclusions

A comparison for the characteristic of two MPCM samples was conducted: one sample was Micronal^®^ DS5008 X while the other was made in our laboratory (M-2). The shell material for both samples is similar in terms of chemical composition. Following SEM observation, it can be concluded that the two samples have similar shape and diameter of about 6 μm, but they have different morphology since Micronal^®^ DS 5008 X capsules were produced as aggregates of many microcapsules. Regarding the thermophysical properties for both samples, their thermal energy storage (TES) capacity were 111.7 J·g^−1^ and 99.3 J·g^−1^ for M-2 and Micronal^®^ DS 5008 X, respectively. The mechanical testing was performed by measuring elastic modulus (*E*), load at displacement (*P_m_*), and displacement at maximum load (*h_m_*) using nano-indentation technique. Different results were obtained for both samples, showing that evaluating the isolated M-2 sample has better mechanical resistance and stiffness. Finally, a comparative evaluation of the VOC’s release at 25 °C, 35 °C, 45 °C, 55 °C, and 65 °C was performed in order to study the volatiles emission from these microcapsules. M-2 microcapsules show better stability with less short-term emission of VOC’s than Micronal^®^ DS 5008 X.
